# A Hidden Struggle: Understanding The Effects Of Bereavement After Caregiving Ends

**DOI:** 10.1093/geroni/igaf122.3313

**Published:** 2025-12-31

**Authors:** C Cassie Heilman, Julie Hicks-Patrick

**Affiliations:** West Virginia University, Morgantown, West Virginia, United States; West Virginia University, Morgantown, West Virginia, United States

## Abstract

Research shows that up to 20% of caregivers of persons with dementia experience an increase in complicated grief and depressive symptoms following the death of the care recipient (Schulz et al., 2006). We used the 2023 BRFSS, which asked caregivers whether their care recipient had died within the past 30 days. We used independent samples t-test to examine mental and physical health differences between recently bereaved (n = 77) and current caregivers (n = 11233). Bereaved caregivers reported more poor mental health days per month compared to their non-bereaved counterparts (M = 9.36 v. 5.88, t(11379) = 5.07, p < .03). Bereaved caregivers reported a lower incidence of lifetime depression than non-bereaved caregivers (16% v. 27%; t(11501) = 28.70, p < .001). However, the two groups did not significantly differ on the number of days of poor physical health (M = 5.34 and 4.65). Bereaved caregivers report poorer mental health than those who are continuing to provide care, illustrating that even after the burden of caring for a loved one has passed, the hardships for the caregiver are not over. Post hoc analyses examine potential moderators of these differences. Given the lack of interventions available to assist caregivers with their grief (Arruda & Paun, 2017), expanding programs to assist recently bereaved caregivers could alleviate mental health challenges among this group.

